# Supplementary material for: A single neuron subset governs a single coactive neuron circuit in Hydra vulgaris, representing a possible ancestral feature of neural evolution
Source: Sci Rep. 2021 May 24;11:10828. doi: 10.1038/s41598-021-89325-x (PMC8144215; doi:10.1038/s41598-021-89325-x)
Supplement: Supplementary file 1 — Supplementary Figures. [file 41598_2021_89325_MOESM1_ESM.pdf]

A single neuron subset governs a single coactive neuron circuit in *Hydra vulgaris*, representing a possible ancestral feature of neural evolution

Yukihiko Noro[1)], Hiroshi Shimizu[1)], Katsuhiko Mineta[1)] and Takashi Gojobori[1)*]

[1)] Computational Biosciences Research Center, King Abdullah University of Science and Technology, Thuwal 23955-6900, Kingdom of Saudi Arabia

Gene#	Group	Gene	Peptide	Expression: Tentacle	Hypostome	Body column: upper	Body column: lower	Peduncle: upper	Peduncle: lower: upper	Peduncle: lower: lower	Basal disk
1	GLWa	*GLWa*	I, II, III, IV	v	v	v	v	v	v	v	v
2	Hym-355	*Hym-355*	Hym-355	v	v	v	v	v	v	v	v
3	RFa	*Prepro A*	I, II, III, IV	v	v	v			v	v	
4		*Prepro B*	I, II		v	v					
5		*Prepro C*	I	v							
6	Hym-176	*Hym-176A*	Hym-176, Hym-357		v	v	v	v	v	v	
7		*Hym-176B*	Hym-357		v	v	v	v			
8		*Hym-176C*	(Hym-176, Hym-357)						v	v	
9		*Hym-176D*	(Hym-176)						v	(v)(*3)	
10		*Hym-176E*	–	v							

Classical subsets	Gene#	New subsets: Tentacle	Hypostome	Body column upper	Body column lower	Peduncle upper	Peduncle lower upper	Peduncle lower lower	Basal disk
I	1+2(*1)	ec3C	ec3C	ec3B	ec3B	ec3B	ec3A	ec3A	ec3A
II	3+5	ec2							
III	3+4		ec4A	ec4B					
IV	6+7(*2)		ec1A	ec1A	ec1A	ec1A			
V	3+6+8+9						ec5	ec5	
VI	3+6+8						ec5	ec5	
VII	10	ec1B							

Fig. S1. Mutually exclusive peptidergic neuron subsets in the ectodermal layer. Upper panel: Neuropeptide genes distinguished by "Gene#" are expressed in neurons located in different regions of *Hydra*. Different gene expression shown in the same color in a given region indicates co-expression in the same neurons. Peptides in parenthesis are similar but not identical. Lower panel: According to the upper panel, mutually exclusive neuron subsets, each of which expresses a different combination of neuropeptide genes, are listed. The "classical subsets," which are defined by classical methods such as immunohistochemistry or in situ hybridization, express genes shown in the column "Gene#." For example, neuron subset V expresses genes #3 (*PreproA*), #6 (*Hym-176A*), #8 (*Hym-176C*), and #9 (*Hym-176D*) in the lower peduncle. A recent single-cell RNA-seq study showed that the classical subset could be further divided into "new subsets." *1) There are some $GLWa^{+}/Hym355^{-}$ neurons in the hypostome. *2) Expression of *Hym-176A* is much less than that of *Hym-176B* in the body column and the upper peduncle. *3) *Hym-176D* is not expressed in this region of strain 105 but expressed in strain AEP.

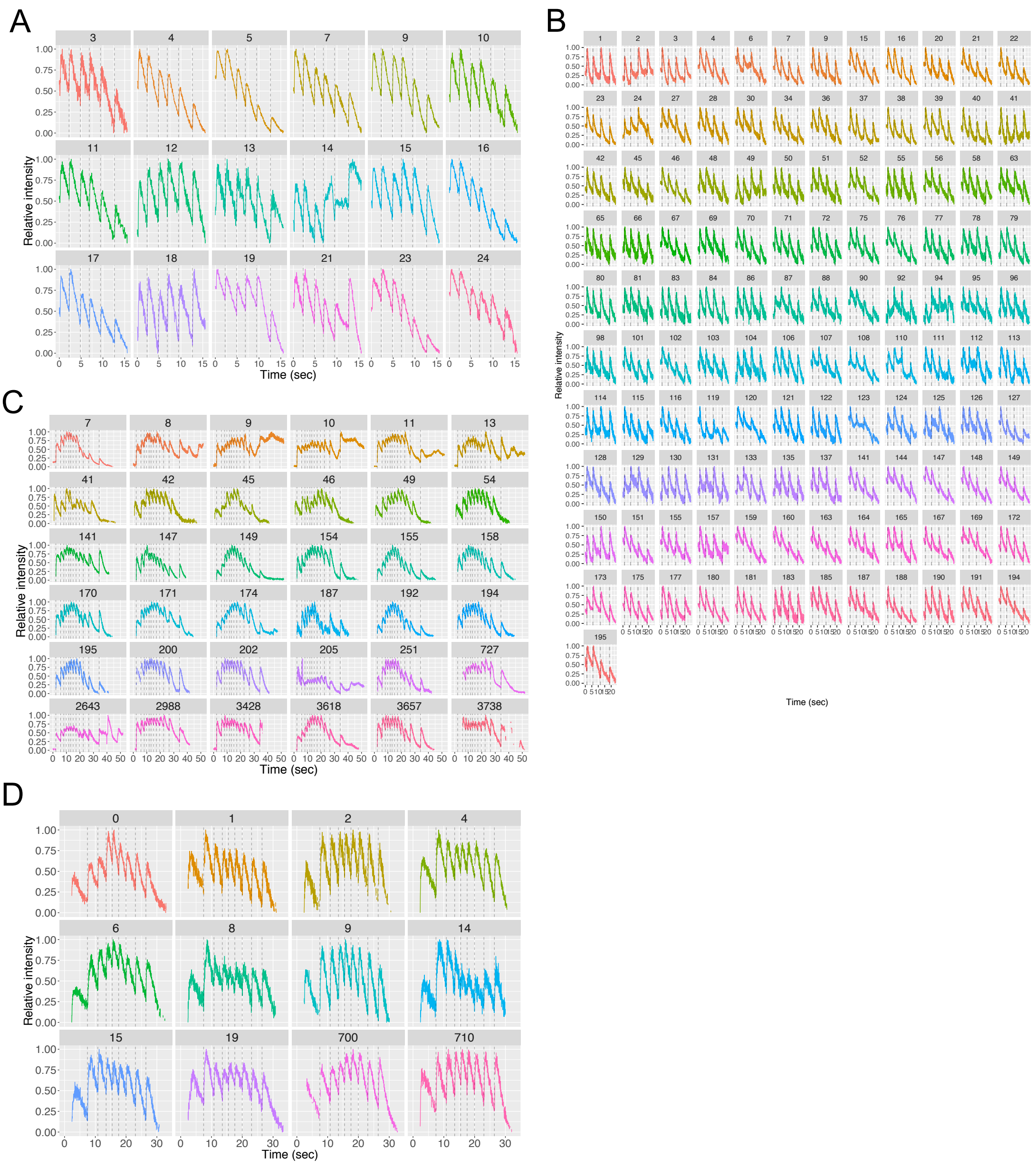

Fig. S2. Normalized neuronal activity in the Hym-176 peptidergic neuron subsets. *Hym-176A*-expressing neuron subset (A). *Hym-176B*-expressing neuron subset (B). *Hym-176C*-expressing neuron subset (C). *Hym-176D*-expressing neuron subset (D). Neuronal activity (normalized relative intensity of GCaMP) with vertical dashed lines indicate the average starting time of excitation of all tested neurons in each subset, as described in Fig. 1. The number in each strip and the color of the excitation profile are also described in Fig. 1.

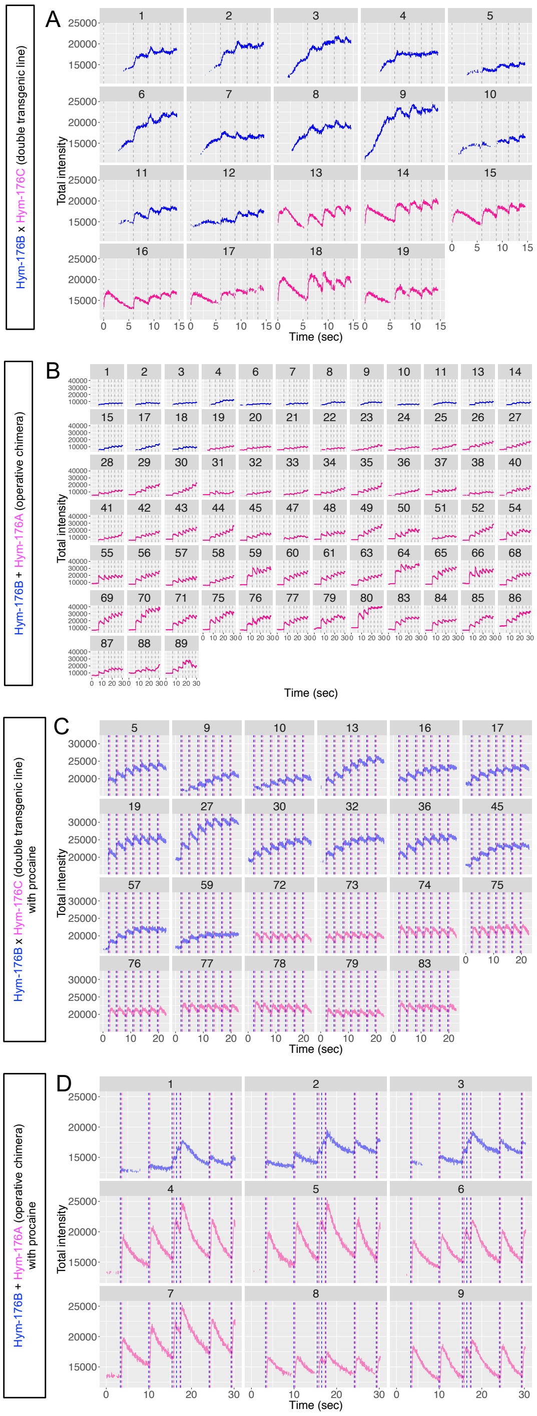

Fig. S3. Unnormalized neuronal activity of the body and the foot neuron circuit. Double transgenic line of Hym-176B::GCaMP and Hym-176C::GCaMP with (C) or without (A) procaine treatment. The operative chimera of Hym-176B::GCaMP and Hym-176A::GCaMP with (D) or without (B) procaine treatment. Unnormalized neuronal activity (total intensity of GCaMP) is plotted as described in Fig. 3B, D, F, H.
