# Supplementary material for: A single neuron subset governs a single coactive neuron circuit in Hydra vulgaris, representing a possible ancestral feature of neural evolution
Source: Sci Rep. 2021 May 24;11:10828. doi: 10.1038/s41598-021-89325-x (PMC8144215; doi:10.1038/s41598-021-89325-x)
Supplement: Supplementary file 7 — Supplementary Legends. [file 41598_2021_89325_MOESM7_ESM.docx]

Movie 1. Hym-176A-expressing neuron subset. Speed: 4 x real-time.

Movie 2. Hym-176B-expressing neuron subset. Speed: 4 x real-time.

Movie 3. Hym-176C-expressing neuron subset. Speed: 4 x real-time.

Movie 4. Hym-176D-expressing neuron subset. Speed: 4 x real-time.

Movie 5. Hym-176A, B, or D-expressing neuron subset. High magnification. Speed: 4 x real-time.

**Movie 6. Hym-176A-expressing neuron subset.** 4 to 8 sec of Supplementary Movie 1 with higher magnification. Speed: 1 x real-time.

Movie 7. Hym-176B-expressing neuron subset. 10 to 14 sec of Supplementary Movie 2 with higher magnification. Speed: 1 x real-time.

Movie 8. Hym-176C-expressing neuron subset. Full length of Supplementary Movie 3 with higher magnification. Speed: 1 x real-time.

Movie 9. Hym-176D-expressing neuron subset. Full length of Supplementary Movie 4 with higher magnification. Speed: 1 x real-time.

Movie 10. Double transgenic line; Hym-176B::GCaMP x Hym-176C::GCaMP. Speed: 1 x real-time.

Movie 11. Operative chimera; Hym-176B::GCaMP + Hym-176A::GCaMP. Speed: 1 x real-time.

Movie 12. Double transgenic line; Hym-176B::GCaMP x Hym-176C::GCaMP treated with procaine. Speed: 1 x real-time.

Movie 13. Operative chimera; Hym-176B::GCaMP + Hym-176A::GCaMP treated with procaine. Speed: 1 x real-time.

Movie 14. Hym-176B::GCaMP transgenic animal treated with procaine. Speed: 1 x real-time.

Movie 15. Hym-176B::GCaMP transgenic animal. Speed: 4 x real-time.
